# Serum Ammonia Levels as a Non-invasive Predictor of the Presence and Severity of Esophageal Varices in Chronic Liver Disease Patients

**DOI:** 10.7759/cureus.39792

**Published:** 2023-05-31

**Authors:** Vivek Kumar, Divyam Kansal, Shyam Chand Chaudhary, K K Gupta, Kauser Usman, Virendra Atam, K K Sawlani, Mahak Lamba, Ajay Kumar, Himanshu Reddy

**Affiliations:** 1 Internal Medicine, King George's Medical University, Lucknow, IND; 2 Internal Medicine, King George’s Medical University, Lucknow, India., Lucknow, IND

**Keywords:** urea, aspartate aminotransferase-to-platelet ratio index (apri score), gastroesophageal varices, serum ammonia, chronic liver disease (cld), gastroenterology and hepatology

## Abstract

Background

Portal hypertension leads to the formation of portosystemic collateral veins, of which esophageal varices (EV) are the most severe complications and have the greatest clinical impact. The possibility of identifying cirrhotic patients with varices by non-invasive tests is appealing, as they can lead to reduced healthcare costs and can be done in resource-limited settings. In this study, we investigated ammonia as a potential non-invasive predictor of EV.

Methods

This was a single-center cross-sectional observational study that was done at a tertiary health care hospital in north India. It included 97 chronic liver disease patients irrespective of etiology after excluding patients with portal vein thrombosis and hepatocellular carcinoma to participate in endoscopic screening for the presence of EV and correlate it with various non-invasive markers like serum ammonia levels, thrombocytopenia and aspartate aminotransferase to platelet ratio index (APRI ). On the basis of endoscopy, enrolled patients were divided into two groups, i.e., group A consisting of large varices (grade III and grade IV) and group B consisting of patients with low-grade varices and no varices (grade II, grade I, and no varices).

Results

This study included 97 patients, out of which 81 patients have varices on endoscopy, and mean serum ammonia levels were found to be significantly higher in cases with varices (135 ±69.70 ) vs. those without varices (94±43) (p value=0.026). Further, on comparing serum ammonia values between patients with large varices (Grade III/IV) (Group A) with a mean value of 176 ± 83 vs. Grade I/II/No varices (Group B) with a mean value of 107±47, which were significantly higher in Group A patients (<0.001). In our study, we also found a correlation between blood urea level as a non-invasive predictor of varices, but no statistically significant relation was found between thrombocytopenia and APRI.

Conclusion

This study found that serum ammonia can be used as a useful marker for the prediction of EV and can also be used to determine the severity of varices. Apart from ammonia, serum urea levels can also prove to be a good non-invasive marker for the prediction of varices although further multicentric studies are warranted to reach this conclusion.

## Introduction

Cirrhosis is the eleventh leading cause of death and the fifteenth leading cause of morbidity, accounting for 2.2% of deaths and 1.5% of disability-adjusted life-years worldwide in 2016. Chronic liver disease caused 1.32 million deaths in 2017, approximately two-thirds among men and one-third among women [1]. However, irrespective of the etiology, the end result is cirrhosis, which has risk factors for developing hepatocellular carcinoma and other complications such as ascites, icterus, gastroesophageal varices, and hepatic encephalopathy [2]. Portal hypertension leads to the formation of portosystemic collateral veins out of which esophageal varices (EV) are the most severe complication and have the greatest clinical impact, which is discovered on endoscopy in up to two-thirds of decompensated cirrhotics. The possibility of identifying cirrhotic patients with EV or other collateral presence by non-invasive means is appealing, in that it could decrease the necessity of endoscopic screening with reduced healthcare costs [3].

Ammonia has been used by some as a marker of encephalopathy but ammonia (NH4) levels are not a good laboratory marker for hepatic encephalopathy, being neither specific nor highly sensitive, and the diagnosis of encephalopathy is mainly clinical (neuropsychiatric) and may be confirmed by instrumental measures such as EEG and psychometric testing. It has been seen that levels of serum ammonia are significantly higher in patients with liver cirrhosis and are gradually elevated further as the severity of liver dysfunction and complications such as esophageal varices increase [4].

In patients with cirrhosis, portosystemic shunts remove most of the ammonia from the portal to systemic circulation, raising the blood ammonia level, which could render blood ammonia measurement useful to reflect the presence of portal hypertension and portosystemic collaterals [5].

Several foreign studies with varying results have discussed how to identify patients with varices using non-invasive or minimally invasive methods, as the development of such methods for varices prediction could reduce the use of upper gastrointestinal endoscopy in variceal screening and provide an alternative way to conventional endoscopic diagnosis [6-8]. Due to conflicting results and the lack of such a study on the Indian population, this study was done to investigate the diagnostic utility of venous ammonia levels, thrombocytopenia, and the aspartate aminotransferase to platelet ratio index (APRI ) as non-invasive markers for the presence and severity of esophageal varices.

## Materials and methods

This cross-sectional observational study was done in the Department of Medicine, KGMU, U.P., Lucknow, India, over a period of one year (Dec 2021-Dec 2022) after approval from King George's Medical University Institutional Ethics Committee (approval number-V PGTSC-11A/F25).

The sample size was calculated using the prevalence of chronic liver disease as 60% as reported by many previous studies [3]. Calculation of the number of subjects was done using Z^2^ X PQ / d^2^ where Z = 1.96 at the 95% confidence interval; p = prevalence, d = allowable error (7% allowable error) = 3.84x15x85/0.072 =0.7744/0.0064 = 96. After taking proper consent, 97 patients fulfilling the inclusion criteria were enrolled with chronic liver disease, irrespective of the etiology on the basis of clinical, biochemical, and imaging assessments as evidenced by ultrasonography and/or Fibro scan with or without liver biopsy were included in the present study. Patients with portal vein thrombosis or hepatocellular carcinoma, those in whom endoscopy is contraindicated, including patients with coagulopathy and altered sensorium, patients with reduced mouth opening, and those not giving proper consent for the procedure were excluded from the study.

All enrolled patients were classified based on the Child-Pugh Score and subjected to a detailed history, examination, routine investigations, and upper gastrointestinal endoscopy.

Patients who were found to have varices on endoscopy patients were divided into two groups, i.e., group A consisting of large varices (grade III and grade IV) and group B consisting of patients with low-grade varices and no varices (grade II, grade I, and no varices).

Blood ammonia levels (venous) were estimated, and samples were sent to the laboratory in ethylenediaminetetraacetic acid (EDTA) vial in the iced container after avoidance of factors that may affect the blood ammonia level (BAL), like exercise, smoking, and tourniquet application. Five ml of venous blood was taken and samples were analyzed within 30 minutes of collection. BAL was estimated by the enzymatic UV method using the glutamate dehydrogenase reaction (GLDH-UV) [9].

The data were entered in Excel sheets (Microsoft® Corp., Redmond, US) and analyzed on Statistical Package for the Social Sciences (SPSS) software version 21.0 (IBM Corp., Armonk, US). All the variables were grouped by measures of central tendency and measures of dispersion.

## Results

**Figure 1:**
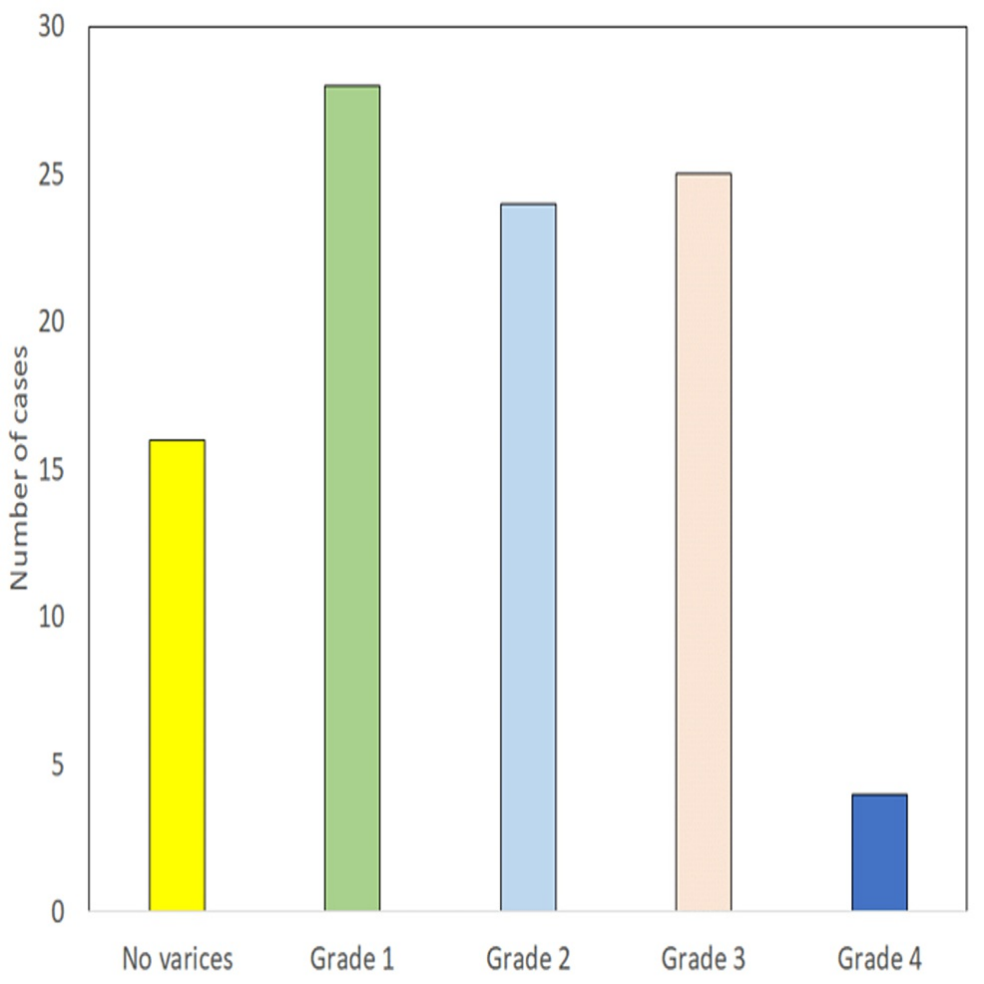
Endoscopy-based distribution of cases

A total of 97 patients with age 14-75 years were included in the study. All the enrolled patients were subjected to endoscopy, which revealed the presence of varices in 81 (83.5%) cases with grade 1, 2, 3, and four varices seen in 28 (28.9%), 24 (24.7%), 25 (25.8%), and four (4.1%) cases, respectively, and 16 (16.5%) cases did not have esophageal varices (Table 1 and Figure 1). The group with esophageal varices was further divided patients into two groups, i.e., Group A with large varices (Grade III/IV) having 29 patients and Group B with low-grade varices and no varices (Grade I/II/No varices) having 68 patients (Table 1 and Figure 1).

**Table 1:** Distribution of cases according to the grade of varices

Finding	No. of cases	Percentage
No varices	16	16.5
Grade 1	28	28.9
Grade 2	24	24.7
Grade 3	25	25.8
Grade 4	4	4.1

**Figure 2:**
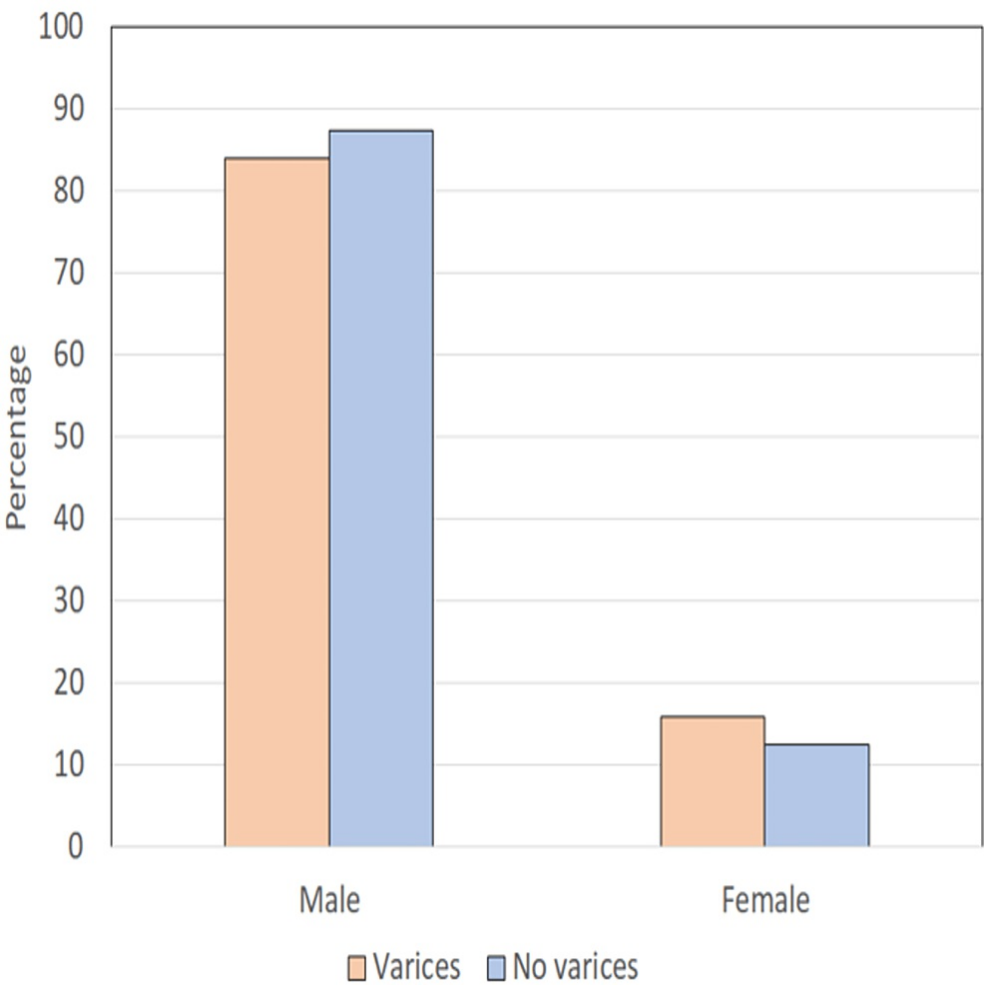
Comparison of gender profile of patients with and without varices

The mean age of patients in our study was 42.07 ±13 years. There was a male preponderance, with 82/97 males (84.5%) and 15 (15.4%) females with a male-to-female ratio of 5.6:1 (Table 2 and Figure 2).

**Table 2:** Characteristics of study respondents with and without esophageal varices SD = standard deviation. p = p value, t = t value, χ² = chi-square test, n = number of subjects

Variable/Parameter	Varices (n=81)	No varices (n=16)	Statistical significance
Mean age ± SD (14-75 years) in years	43.09±12.05 (16-75)	41.44±14.52 (14-60)	t=0.483; p=0.630
Male: Female	68 (84%): 13 (16.0%)	14 (87.5%): 2 (12.5%)	χ²^=^0.129; p=0.720

**Figure 3:**
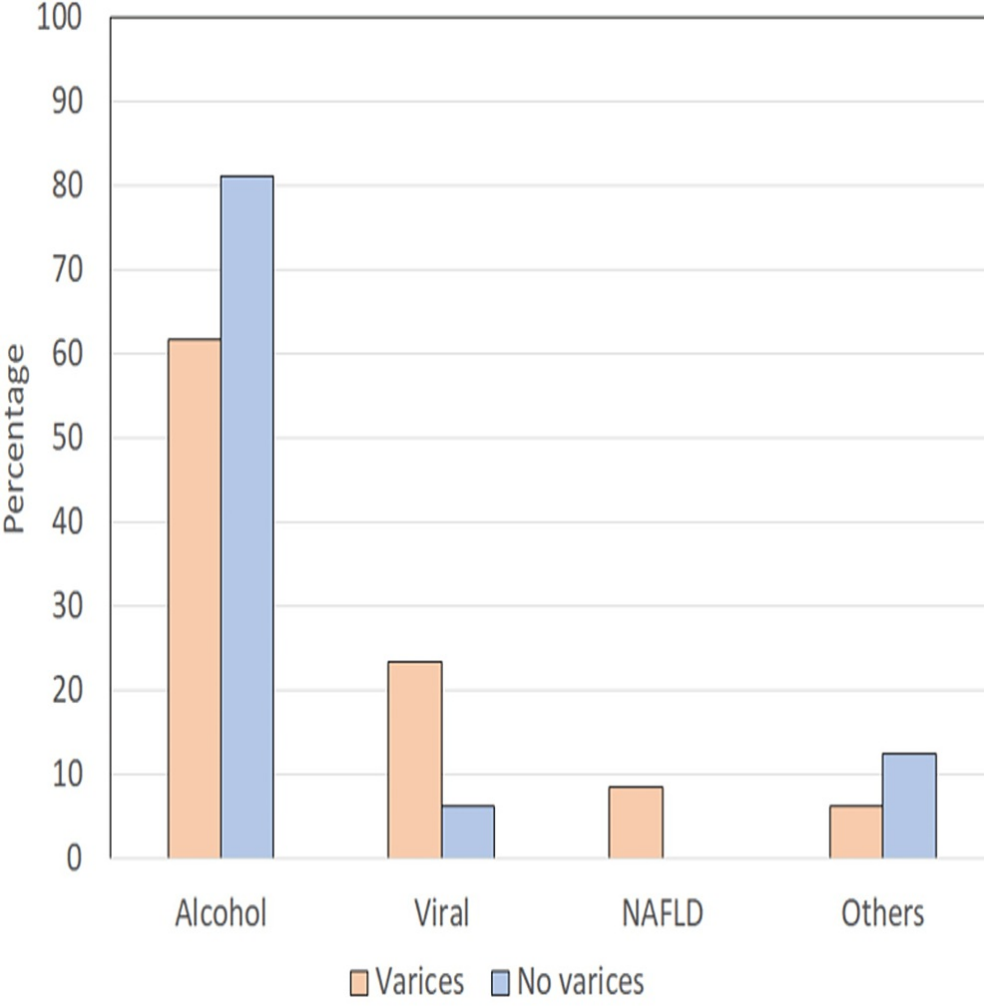
Distribution of patients according to etiological profile NAFLD = non-alcoholic fatty liver disease, n = number of cases, viral = hepatitis B and C

In our study, the most common etiology of chronic liver disease was alcohol, with 50 cases (61.7%); followed by viral hepatitis (B>C), with 19 (23.5%) cases; followed by non-alcoholic steatohepatitis (NASH) with seven (8.6%) cases and five cryptogenic cases (6.2%) (Table 3 and Figure 3).

**Table 3:** Comparison of the etiological profile of cases with and without esophageal varices NASH = non-alcoholic steatohepatitis, n = number of cases, viral = hepatitis B and C

Etiology	Varices (n=81)	No varices (n=16)
Alcohol	50 (61.7%)	13 (81.3%)
Viral	19 (23.5%)	1 (6.3%)
NASH	7 (8.6%)	0 (0%)
Cryptogenic or unknown cause	5 (6.2%)	2 (12.5%)

**Figure 4:**
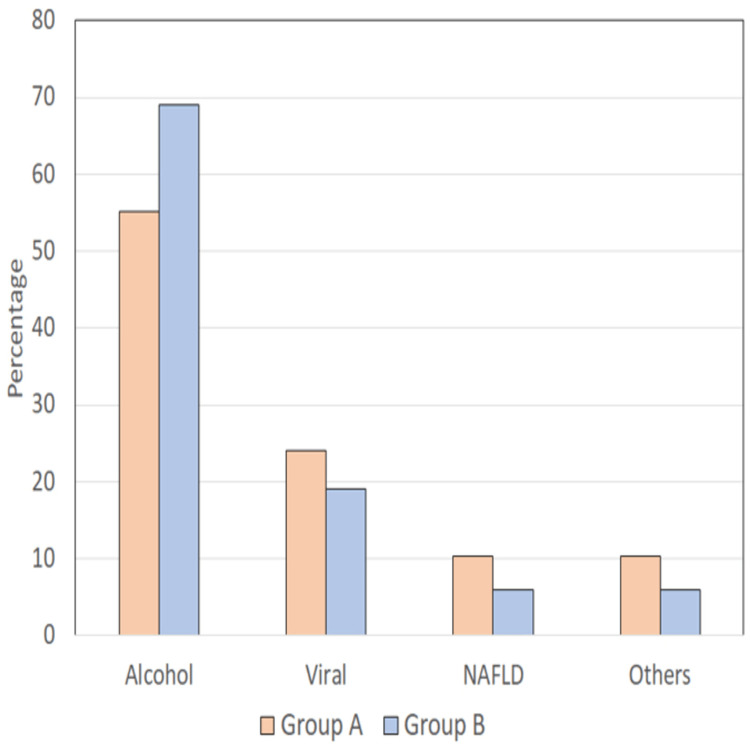
Comparison of etiological profile between large varices (Grade III/IV) (Group A) vs (Grade I/II/No varices) (Group B) NAFLD = non-alcoholic fatty liver disease, n = number of cases, viral = hepatitis B and C

On comparison of patient profiles between Group A large varices (Grade III/IV) vs Group B (Grade I/II/No varices), it was found that the majority of patients had alcohol as the etiology although the proportion of those having other than alcoholic etiology was higher in high-grade varices (44.8%) as compared to that in low-grade varices (30.9%) yet this difference was not significant statistically (p=0.577) (Table 4 and Figure 4).

**Table 4:** Comparison of etiological profiles of large varices (Grade III/IV) (Group A) vs (Grade I/II/No varices) (Group B) NAFLD = non-alcoholic fatty liver disease, n = number of cases, viral = hepatitis B and C

Etiology	Group A (n=29)		Group B (n=68)	
	No.	%	No.	%
Alcohol	18	55.2	47	69.1
Viral	7	24.1	13	19.1
NAFLD	3	10.3	4	5.9
Others	3	10.3	4	5.9

**Figure 5:**
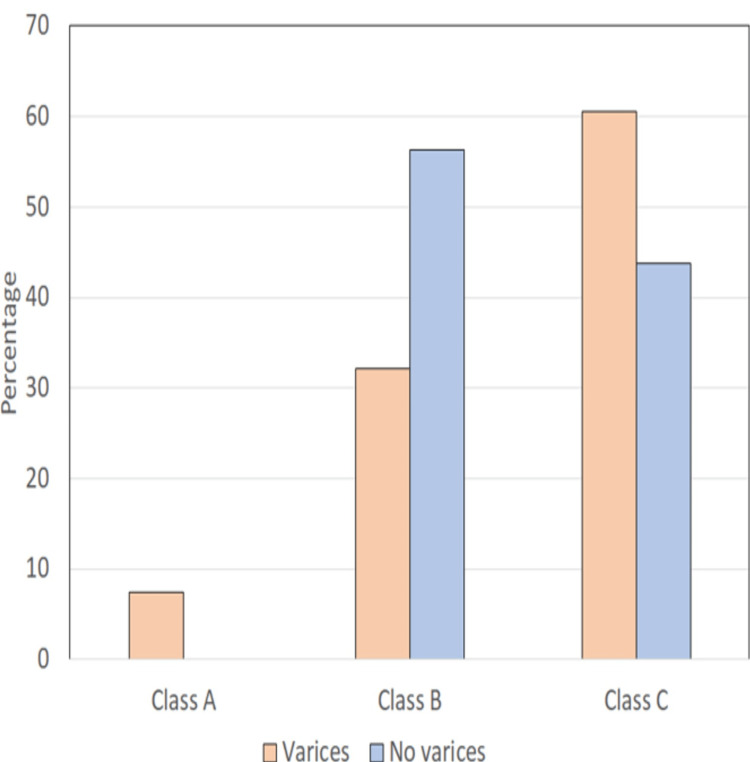
Distribution of cases with and without varices according to Child-Turcotte-Pugh (CTP) classification

Patients were classified based on the Child-Turcotte-Pugh (CTP) class, the majority of patients, i.e., 49 (60.5%) belonged to Class C, followed by 26 patients (32.1%) in Class B, and only six (7.4%) in Class A (Table 5 and Figure 5).

**Table 5:** Distribution of cases with and without varices according to the Child-Turcotte-Pugh (CTP) classification n = number of cases

Child-Pugh class	Varices (n=81)	No varices (n=16)
Class A (5-6 points)	6 (7.4%)	0
Class B (7-9 points)	26 (32.1%)	9 (56.3%)
Class C (10-15 points)	49 (60.5%)	7 (43.8%)

When various biochemical and coagulation parameters were analyzed, there was a statistically significant association between the presence of varices and ammonia levels. Serum urea levels and prothrombin time also have a statistically significant positive association with the presence of varices (Table 5).

**Figure 6:**
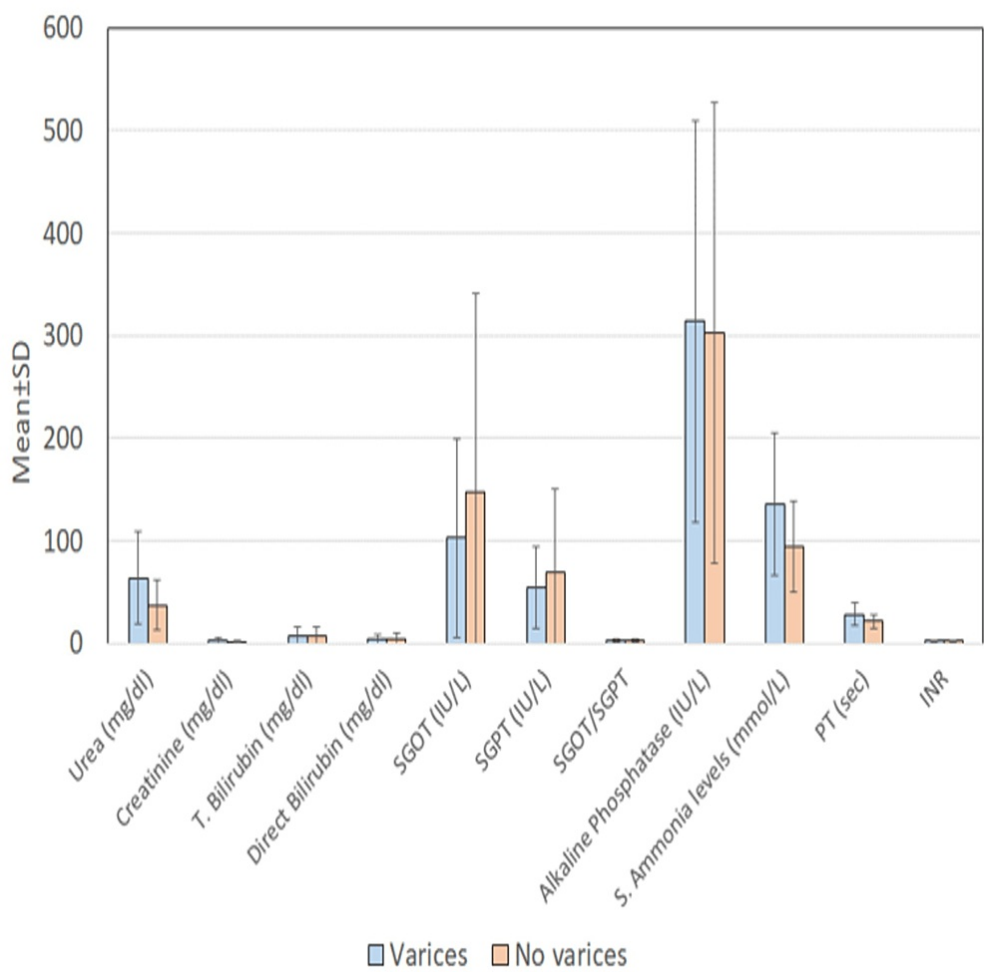
Comparison of biochemical and coagulation parameters between cases with and without esophageal varices (EV) SD = standard deviation, T. bilirubin = total bilirubin, SGOT = serum glutamic oxaloacetic transaminase, SGPT = serum glutamate pyruvate transaminase, PT = prothrombin time, INR = international normalized ratio

Mean serum ammonia levels were found to be significantly higher in cases with varices (135±69.7) mmol/L vs those without varices (94±43) mmol/L (p value=0.026) and on comparing serum ammonia values between patients with large varices (Grade III/IV) (Group A) with mean value (176±83) mmol/L vs. (Grade I/II/No varices) (Group B) with a mean value of 107±47 mmol/L, which were significantly higher in Group A patients(<0.001) (Tables 5, 6 and Figures 5, 6).

**Table 6:** Comparison of biochemical and coagulation parameters between cases with and without esophageal varices N = number of cases, SD = standard deviation, p = p value, t = t test value, T. bilirubin = total bilirubin, SGOT = serum glutamic oxaloacetic transaminase, SGPT = serum glutamate pyruvate transaminase, PT = prothrombin time, INR = international normalized ratio

Variable/Parameter	Varices (n=81)		No varices (n=16)		Statistical significance	
	Mean	SD	Mean	SD	‘t’	‘p’
Urea (mg/dl)	63.63	45.6	37.02	24.5	2.194	0.031
Creatinine (mg/dl)	2.42	3.1	1.44	0.9	1.200	0.233
T. Bilirubin (mg/dl)	6.99	8.1	6.79	8.7	0.087	0.931
Direct Bilirubin (mg/dl)	4.08	4.9	4.00	5.6	0.057	0.955
SGOT (IU/L)	102.22	96.9	146.96	194.3	-1.388	0.168
SGPT (IU/L)	54.03	40.3	68.86	80.8	-1.108	0.271
SGOT/SGPT	2.08	1.1	2.10	1.3	-0.055	0.956
Alkaline Phosphatase (IU/L)	313.97	195.7	302.32	225.0	0.212	0.832
S. Ammonia levels (mmol/L)	135.07	69.7	94.01	43.9	2.264	0.026
PT (sec)	28.21	11.1	21.04	7.0	1.978	0.051
INR	2.18	0.8	1.77	0.6	1.603	0.113

Mean values of blood urea levels in patients with varices (63 ± 45 ) mg/dl vs without varices (37 ±24) mg/dl (p value-0.031) and on comparing serum urea values between patients with Large varices (Grade III/IV) (Group A) with a mean value of 61.46± 50.1 mg/dl vs Grade I/II/No varices) (Group B) with a mean value of 58.30±41.0 mg/dl, which was higher in Group A patients but the correlation was not significant statistically(p value=0.750) (Tables 5, 6 and Figures 5, 6).

**Figure 7:**
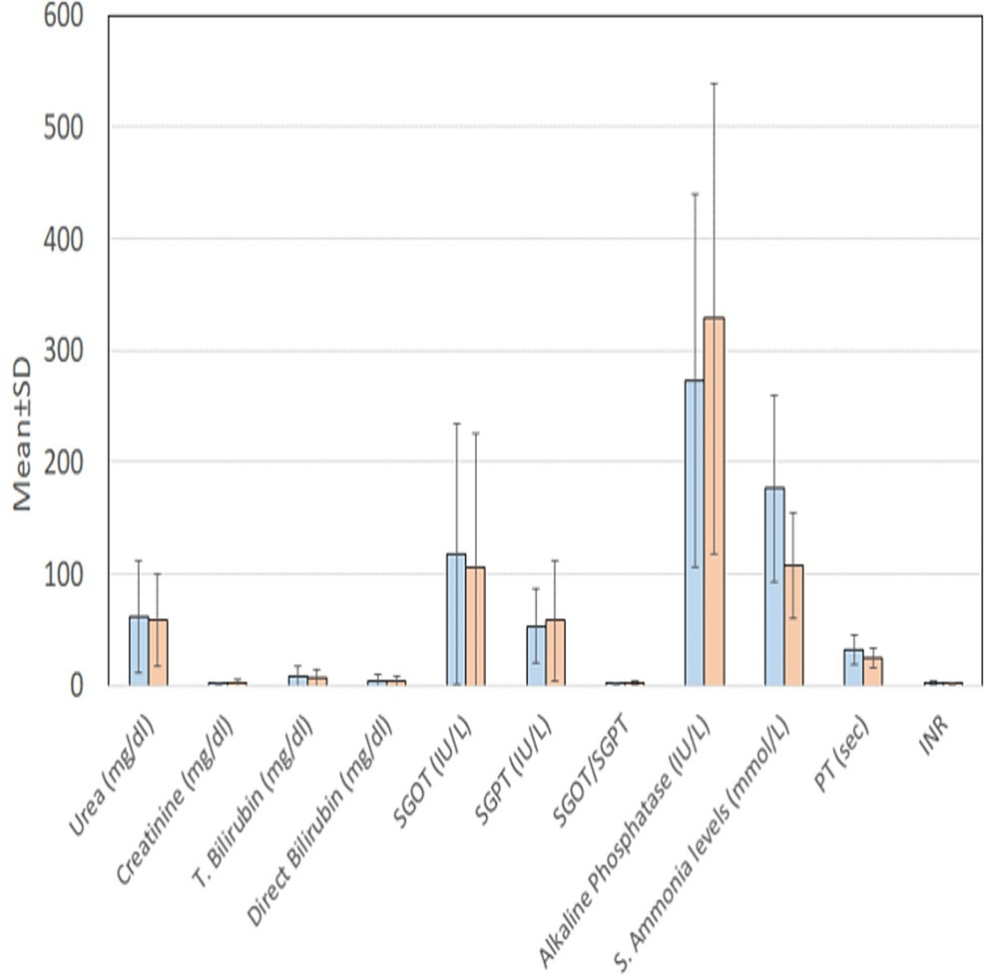
Comparison of biochemical and coagulation parameters between patients with large varices (Grade III/IV) (Group A) vs Grade I/II/No varices (Group B) SD = standard deviation, T. bilirubin = total bilirubin, SGPT = serum glutamate pyruvate transaminase, PT = prothrombin time, INR = international normalized ratio

The mean value of Prothrombin time was (28.21 + 11.5) sec in the group with varices as compared to the group without varices (21.04 +7.0) sec but was marginally insignificant (p value-0.051) although on comparing serum prothrombin time values between patients with Large varices (Grade III/IV) (Group A) with a mean value of 31.63± 13.2 sec vs. Grade I/II/No varices (Group B) with a mean value of 25.21±8.9 sec, which was significantly higher in Group A patients (<0.009) (Tables 6, 7 and Figures 6, 7).

**Table 7:** Comparison of biochemical and coagulation parameters between patients with large varices (Grade III/IV) (Group A) vs Grade I/II/No varices (Group B) n = number of cases, SD = standard deviation, p = p value, t = t test value, T. bilirubin = total bilirubin, SGPT = serum glutamate pyruvate transaminase, PT = prothrombin time, INR = international normalized ratio

Parameter	Group A (n=29)		Group B (n=68)		Statistical significance	
	Mean	SD	Mean	SD	‘t’	‘p’
Urea (mg/dl)	61.46	50.09	58.30	41.08	0.320	0.750
Creatinine (mg/dl)	1.82	1.31	2.45	3.36	-0.956	0.342
T. bilirubin (mg/dl)	7.83	9.20	6.58	7.70	0.689	0.492
Direct bilirubin (mg/dl)	4.47	5.81	3.89	4.70	0.516	0.607
SGOT (IU/L)	117.66	117.29	106.17	119.54	0.436	0.664
SGPT (IU/L)	52.99	33.18	57.96	54.54	-0.455	0.650
SGOT/SGPT	2.14	0.88	2.06	1.25	0.279	0.781
Alkaline phosphatase (IU/L)	272.66	166.81	328.85	211.00	-1.273	0.206
S. ammonia levels (mmol/L)	176.27	83.19	107.84	47.50	5.120	<0.001
PT (sec)	31.63	13.27	25.21	8.96	2.659	0.009
INR	2.33	0.91	2.04	0.68	1.678	0.097

**Figure 8:**
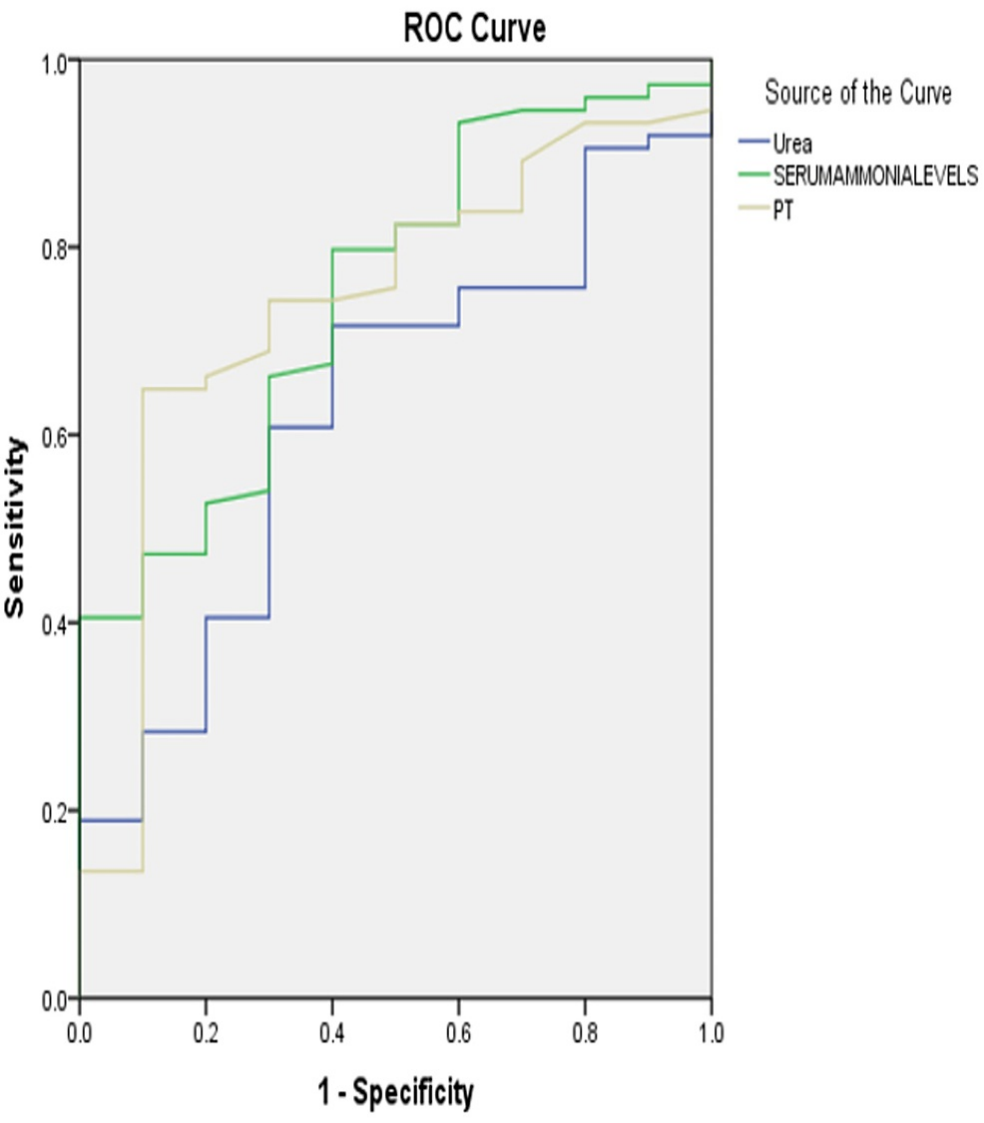
ROC analysis for the derivation of cut-off values of different parameters for the prediction of large varices ROC = receiver operator curve, PT = prothrombin test

On receiver operator curve (ROC) analysis for the derivation of the cut-off values of serum urea, serum ammonia, and prothrombin time for prediction of the presence varices, the area under the curve values ranged from 0.63±0.09 (serum urea) to 0.75±0.07 (serum ammonia). The highest Youden index (J-value) was obtained for prothrombin time (J=0.549), which was found to be 64.9% sensitive and 90% specific at a cut-off value of >23.50 sec. The optimized cut-off value derived for serum ammonia was >147 mmol/L, which was found to be 59.5% sensitive and 100% specific. Serum urea had a minimum optimized discriminant value (J=0.316 only) and had a sensitivity of 71.6% and specificity of 60% at a cut-off of >31.95 mg/dl (Table 8 and Figure 8).

**Table 8:** Receiver operator curve (ROC) analysis for the derivation of the cut-off values of different parameters for the prediction of varices AUC = area under curve, SE = standard error

Parameter	AUC±SE (p value)	Cut-off value (Youden Index-J)	Projected sensitivity	Projected specificity
Serum urea (mg/dl)	0.63±0.09 (0.199)	>31.95 (J=0.316)	71.6%	60%
Serum ammonia (mmol/L)	0.75±0.07 (p=0.010)	>147 (J=0.405)	59.5%	100%
Prothrombin time (sec)	0.74±0.08 (p=0.014)	>23.50 (J=0.549)	64.9%	90%

APRI) was also calculated for all enrolled patients and mean APRI was higher in cases with varices (2.91±2.9) as compared to that in cases without varices (2.37±1.6), however, this difference was not significant statistically (p=0.516).

## Discussion

Many previous studies have documented the good predictive value of various non-endoscopic variables for the presence or absence of varices, but available data in our part of the country are limited. So, we thought of determining serum ammonia levels and their correlation with portosystemic shunts in Indian patients.

In our study, the mean age of patients was 42.07±13 years, ranging from 14 to 75 years So, the majority of patients were of middle age, ranging between 41 to 60 years. The result of our study regarding age was in concordance with the studies done by Mohamed Aboushady et al. [9] and Baig et al. [10], who evaluated non-invasive parameters for detecting esophageal varices (EVs) in liver cirrhosis, The mean age of patients in their study was 51 years, ranging from 20 to 80 years. So in most studies, the middle-aged populations between 40 and 60 years were affected, but they were discordant with Tarantino et al. [3] in which the mean age of patients was age 65 years, which can be due to different regional epidemiology and risk factors.

In our study, there was a male preponderance, with 82/97 (84.5%) being males and 15 (15.4%) being females, with a male-to-female ratio of 5.6:1. These results regarding gender were consistent with those reported by Hassan et al. [11] and Montasser et al. [7] with a male-to-female ratio of 5.6:1 and 3:1, respectively. The reason for male preponderance might be related to the higher percentage of alcohol consumption and high-risk behavior for contracting hepatitis B and C.

The most common etiology in our study was chronic alcoholic liver disease, with 63/97 (64.9%) patients followed by viral hepatitis (HEP B and C)-related chronic liver disease 20(20.6%) followed by NASH, which constituted seven (7.2%), and others (cryptogenic or unknown cause) constituted seven (7.2%) as the cause of cirrhosis. Our results regarding etiology were consistent with studies done by Baig et al. [10] and Sharma et al. [12] to evaluate epidemiological data on the etiological profile of cirrhosis of the liver, where alcohol was the leading cause of cirrhosis followed by hepatitis B and C. So, in most Indian studies, alcohol is the most common etiology.

To assess the severity, patients were classified into three groups according to the Child-Pugh criteria. The maximum number of patients were in group III (n=49) followed by group II (n=26) and group I (n=6). Most of the patients enrolled in our study had advanced cirrhosis, Majority of patients with varices were in Class C (60.5%) whereas most cases without varices were in Class B (56.3%) and only 6 (7.4%) of patients with varices and without varices were in Class A. Due to the tertiary care center, most of the patients were CTP-C and we did not find any significant correlation between the Child-Pugh class and the presence of varices.

Regarding biochemical and coagulation parameters, in our study, we found that serum ammonia was significantly higher in cases with varices as compared to those in cases without varices, with a mean value of 135±69.70 in patients with varices and a mean value of 94±43 in patients without varices (p value=0.026). Also, when comparing serum ammonia levels, they were found to be significantly higher in Group A consisting of large varices (Grade III and Grade IV) as compared to that in Group B consisting of patients with low-grade varices and no varices (Grade II, Grade I, and no varices) with a mean value of 176± 83 and 107±47, respectively,(p value=<0.001).

In the current work, it was also crucial to determine a cut-off value for serum ammonia to identify patients with and without varices. On ROC analysis, it was found that serum ammonia level > 147 μg/dL can predict the presence of esophageal varices. These findings were in concordance with a study done by Montasser et al. [7] who found that a serum ammonia level of > 133 μg/dL can predict the presence of esophageal varices. Other studies like Tarantino et al. [3], Khondaker et al. [5], Darweesh et al. [13], and Elzeftawy et al. [14] reported ammonia levels at a cut-off of 42 µmol/L, ≥63 µmol/L, 82 µmol/L, and 77 µmol/L, respectively, in detecting EV, suggesting its usefulness in identifying patients with large varices who need endoscopies. Also, Lashin et al. [15] concluded that ammonia levels at a cut-off value of 54.84 µmol/L can detect varices. Contrary to this, the study of Ramzy et al. [6] concluded that ammonia alone cannot predict the presence or the grade of EV but its endorsement within a significant prediction model can help restrict the use of endoscopic screening in patients with a high probability of EV presence.

There were many studies supporting the fact that blood ammonia levels can be used as a non-invasive predictor of esophageal varices, but there was a difference in cut-off values in all studies. This can be because serum ammonia levels are affected by many external confounding factors like collection, handling, storage, and analysis of blood samples, which are all potential sources of error. Recommendations ought to be made on the collection and processing of blood samples, for it is by standardization and rigid adherence to these techniques that the reliability of the test results will be improved.

Other than ammonia mean values for prothrombin time, with a mean value of 28.21±11.5 and 21.04±7.0, in a group with esophageal varices and without varices, respectively, but this correlation was marginally insignificant (p value-0.051). But mean values of urea in a patient with varices and without varices were 63±45 and 37±24, respectively, showing a statistically significant correlation (p value-0.031). These findings were in concordance with a study done by Chopra et al. [16] on blood urea concentration as an independent marker for the prediction of positive endoscopic findings in presumed upper gastrointestinal bleeding showed that urea may be used as a surrogate marker for the prediction of upper gastrointestinal bleed. But when we compared serum urea levels between large varices (Grade III/IV) (Group A) vs Grade I/II/No varices) (Group B), there was no statistically significant difference between the two groups, signifying that urea levels can be used to predict the presence of varices but cannot discriminate between groups with high-grade vs low-grade varices.

In our study, we found that both groups (with varices and without varices) had thrombocytopenia with a mean platelet count of 1.14 lac/cumm and 1.37 lac/cumm in the group with varices and without varices, respectively. Although thrombocytopenia was more common in the group with varices, the correlation was not statistically significant and contrasted with studies by Tarantino et al. [3] and Zaman et al. [17], which showed a correlation between the severity of thrombocytopenia with the presence of varices. The difference may be explained by the fact that most patients in our study were of advanced cirrhosis, i.e., CTP Class C, whereas in their study, most patients were CTP Class A and early CTP Class B. We also didn’t find any statistically significant correlation with APRI because platelet count is an important parameter in calculating APRI scores.

There were some limitations of our study because being a study from a single tertiary care center, most of the patients were in an advanced stage of liver fibrosis, i.e., CTP Class B and C and blood ammonia determination suffers from some limits in its measurements like blood ammonia levels vary with diet and body mass index.

Further studies with larger sample sizes are warranted to estimate the correlation between serum ammonia levels and esophageal varices.

## Conclusions

Serum ammonia can be used as a useful marker for the prediction of esophageal varices and can also be used to determine the severity of varices; therefore, it could be used for cirrhotic patients in resource-limited settings or to prioritize high-risk patients to undergo early endoscopy. Apart from ammonia levels, serum urea levels and prothrombin time can also prove to be a good non-invasive marker for the prediction of portosystemic collaterals although further multicentric studies are warranted to reach this conclusion.
